# Reduction of Afterdrop by Using Active External Warming During Treatment of Accidental Hypothermia—A Randomized, Crossover Trial

**DOI:** 10.1111/aas.70162

**Published:** 2025-12-01

**Authors:** Sigurd Mydske, Ane M. Helland, Nicola Borasio, Guttorm Brattebø, Øyvind Østerås, Øystein Wiggen, Jörg Assmus, Giacomo Strapazzon, Øyvind Thomassen

**Affiliations:** ^1^ Department of Clinical Medicine University of Bergen Bergen Norway; ^2^ Department of Anesthesia & Intensive Care Haukeland University Hospital Bergen Norway; ^3^ Mountain Medicine Research Group, The Norwegian Air Ambulance Foundation Bergen Norway; ^4^ Institute of Mountain Emergency Medicine, Eurac Research Bolzano Italy; ^5^ Sports and Exercise Medicine Division, Department of Medicine University of Padova Padova Italy; ^6^ Norwegian National Advisory Unit on Emergency Medical Communication Haukeland University Hospital Bergen Norway; ^7^ SINTEF Digital, Health Research Trondheim Norway

## Abstract

**Introduction:**

This study investigates the impact of active external warming on afterdrop in simulated accidental hypothermia, compared to the effect of passive warming measures.

**Methods:**

This study used data from a randomized crossover field study of experimental hypothermia. Hypothermia was induced in 11 healthy volunteers by pharmacological inhibition of endogenous thermoregulation and exposure to −2°C ambient air in a glacial ice tunnel, wearing wet clothes for a maximum of 2 h, or until their esophageal temperature reached 35°C. Esophageal temperature was measured using a transnasal probe. After cooling, wet clothing was removed and the participants were placed in a hypothermia wrap system for 1 h, with three different sources of active warming applied to the participants in the intervention scenario. After 1 h, the participants were walked with minimal support for balance out of the ice tunnel to a warm tent to recover. The 10 min after the end of the rewarming phase was defined as the afterdrop phase.

**Results:**

Most participants experienced a small drop in temperature during the wrapping phase between the cooling and rewarming phases, followed by a second larger afterdrop while walking out of the ice tunnel. The afterdrop was smaller in the actively rewarmed group, with a mean drop of 0.3°C (min 0.0°C, max 1.2°C) compared to 0.7°C (min 0.0°C, max 1.6°C) in the passively rewarmed group.

**Conclusions:**

Adding active external warming reduces afterdrop compared with using only passive external warming measures in non‐shivering individuals. These results may increase understanding of both the afterdrop phenomenon and heat distribution in the body during rewarming.

**Editorial Comment:**

This study assessed temperature afterdrop in controlled hypothermia in healthy volunteers who were treated with active external rewarming or passive rewarming. These findings provide detailed insight into how temperature redistribution can occur during rewarming, at least in healthy cases.

## 1 Introduction

The human body is a homeothermic organism that regulates its internal body temperature by physiological and behavioral actions, maintaining a steady state of heat loss and thermogenesis between core and peripheral tissues [1]. At rest, heat production occurs mainly in core tissues and in skeletal muscle, and heat flows through tissues before being dissipated from the skin [2].

Patients with accidental hypothermia have often been exposed to cold ambient air or water [3]. Heat loss from the skin to the ambient environment, combined with peripheral vasoconstriction, causes a greater temperature decrease in the peripheral tissues relative to core tissues. When cooling stops (e.g., when the victim is rescued and insulated from the cold environment), core temperature may continue to drop until a stable thermal gradient between the different thermal compartments of the body is re‐established [4, 5, 6]. This physiological phenomenon is called “afterdrop.”

The roles of the conductive and convective mechanisms of afterdrop have been debated [7, 8, 9]. A conductive mechanism means that a stable thermal gradient will be re‐established via tissue heat flux [8]. The convective mechanism suggests that the circulating blood volume will act as a heat vector, flowing between various tissue compartments, and thereby distributing heat from the core to the periphery more quickly [7]. Both mechanisms contribute to afterdrop, but the clinical importance of each mechanism is likely dependent on several factors, such as the core temperature or the amount of movement to which the patient is subjected [4].

An additional drop in temperature may have clinical implications during rescue, with more severe complications expected with decreasing core temperatures (e.g., arrhythmias or even cardiac arrest) [6, 10, 11], especially in patients with moderate and severe accidental hypothermia. Evidence suggests that alert patients who have been exposed to cold and are categorized as having mild hypothermia may safely be mobilized [12], but some conflicting opinions are present in clinical recommendations [2]. If active rewarming reduces the afterdrop, one can potentially mitigate the risk of severe complications during mobilization. We hypothesized that the addition of active external warming may contribute to reduced afterdrop. This study aimed to investigate how the addition of active external warming impacts the afterdrop in experimental accidental hypothermia compared to the effect of using only passive warming measures in research participants using inhibition of shivering.

## 2 Methods

The Norwegian Regional Ethics Committee for Medical and Health Research (2024/714469) REK South‐East C and the Data Protection Officer of Haukeland University Hospital approved the original trial, and the trial was registered at ClinicalTrials.gov (NCT 06342726).

This experiment was conducted in May and June 2024 as part of a larger study investigating the effects of active external warming on esophageal temperature rewarming rate (NCT05779722). Participants were subjected to a cooling phase in an ice tunnel for a maximum of 2 h, or until they reached an esophageal temperature of 35°C using a protocol for pharmacological inhibition of endogenous thermoregulation [13]. The cooling phase was followed by a 1‐h rewarming phase, during which participants were rewarmed using either passive or a combination of active and passive warming techniques. Thereafter, esophageal temperature changes were evaluated when the participants were assisted to a standing position before they walked with minimal support for balance, approximately 70 m out from the ice tunnel. The esophageal temperature of the participants was recorded while they were walking, measuring afterdrop. The participants were subjected to a mandatory 5‐day wash‐out period between exposures to ensure complete elimination of the medication, before repeating the opposite scenario, serving as their own control.

### 2.1 Selection of Participants

A preliminary power analysis for the original trial estimated a required sample size of nine participants in each group to achieve a power of 0.9 for a two‐sided *t*‐test at a significance level of 0.05, assuming a minimal clinically relevant difference in core temperature of 0.3°C and a standard deviation of 0.2. Twelve healthy participants aged 18–50 years were recruited through an open invitation aimed at both professional and volunteer search and rescue services/organizations, including medical students and paramedics. Baseline characteristics for the participants may be found in Table 1, and a complete list of inclusion and exclusion criteria can be found in the Supplemental files.

**TABLE 1:** Baseline characteristics: baseline characteristics of the research participants. BMI required to be between 18.5 and 30 kg/m^2^.

Rewarming method in the first of two runs^a^		
	Passive *n* = 6	Active *n* = 5
Age (years)^b^	24.5 (21, 54)	25 (22, 32)
Sex (female)^c^	4 (75%)	3 (60%)
BMI^b^	25.8 (20.4, 29.6)	24.9 (20.8, 26.6)

^a^ Opposite scenarios completed 5 days later.

^b^ Median (min, max).

^c^ *N* (%).

### 2.2 Study Setting

The experiments were performed using an ice tunnel at *Klimapark2469* in Lom, Norway, as a climate chamber. The tunnel is a 70‐m long, excavated cave in a glacier located at approximately 1850 m above sea level, with a constant temperature of around −2°C and no wind. A heated recovery tent (NorLense SWIFT System 5 × 6 m) was erected outside the ice tunnel, where the participants could recover safely after all of the experiments were concluded.

### 2.3 Study Design and Experimental Procedure

In this crossover trial, participants were randomized by draw to either the passive or active warming scenario on the first day of the experiments, followed by the opposite scenario 5 days later. All participants had a light meal before the experiments, and abstained from alcohol, caffeine and physical exercise before the experiments. During cold exposure, participants were placed in a supine position on a 14‐mm thick Mammut Bamse Extreme sleeping pad (Mammut Sports Group, Seon, Switzerland; R‐value 1.9) while wearing soaking, but not dripping, wet, single‐layer cotton clothing. The clothing was soaked in water shortly before the experiments. At the end of the cooling phase, wet clothing was quickly removed, and the participants were carefully log‐rolled into a hypothermia wrap system with a vapor barrier (ASAP JONA 200, ASAP Norway, Skien, Norway), a large sleeping bag (Carinthia Defence 6 G‐LOFT 435 g/m^2^, Goldeck Textil GmbH, Seeboden, Austria), and a mountain quilt (Jerven Extreme Primaloft 170 g/m^2^, Jerven AS, Odda, Norway) [5, 14]. An additional insulating sleeping pad (Z‐lite, Therm‐a‐Rest, Seattle, WA, USA, *R*‐value 2.0) was placed underneath the participants during the rewarming phase.

For participants in the group receiving active external warming, in addition to the hypothermia wrap system, an electric heating blanket was placed directly on the skin of the torso (PAX Warming blanket, X‐CEN‐TEK GmbH, Germany), a chemical heating blanket was placed outside the vapor barrier on the lower body (Ready Heat II, TechTrade, Orlando, FL, USA), and a heated balaclava covered by a hat was fitted (HAT Hypothermia Active Treatment, Northerm Medical, Minitech AS, Ridabu, Norway).

The completion of the wrapping marked the beginning of the 1‐h rewarming phase, and the period investigated in this study was set to “end of rewarming phase” + 10 min, to investigate the afterdrop.

### 2.4 Inhibition of Thermoregulation

Helland et al. developed a protocol for suppressing endogenous thermoregulation in healthy participants to induce experimental hypothermia [13]. Thermoregulatory suppression is achieved mainly through inhibition of shivering, and this protocol was applied in our study. All participants received an oral dose of 30 mg of buspirone 1 h before the start of the experiment. Immediately before the start of the experiment, each participant was administered a dose of meperidine 1 mg/kg intravenously, dividing the bolus dose into five aliquots administered every 2 min for 10 min. During the 3‐h experimental period, maintenance boluses (0.5 mg/kg) were administered every 25 min. To reduce nausea, Ondansetron 4 mg was administered intravenously immediately before the first dose of meperidine. One additional dose of 4 mg of intravenous ondansetron was available to participants during or after the experiment if needed.

### 2.5 Measurements and Outcomes

The esophageal temperature was measured using a transnasal esophageal probe (Type ER400‐9, Smiths Medical, London, UK. Accuracy ±0.1°C), which was positioned before the start of the experiments, and placement was verified using a flexible scope if misplacement was suspected. Esophageal temperature was recorded once every minute using a Corpuls3 multimonitor (Corpuls, GS Elektromedizinische Geräte G. Stemple GmbH, Germany). This device also recorded a 4‐lead continuous ECG, finger SpO_2_ measurements, and non‐invasive blood pressure monitoring, which was measured every 15 min.

### 2.6 Statistical Analysis

The primary outcome was the difference in afterdrop during  the first 10 min after the end of the rewarming phase between the intervention and control groups. Because the groups exhibited baseline differences, statistical tests were not performed for direct comparison. Instead, a 95% confidence interval was reported to provide estimates of effect size and precision. R 4.4.0 was used for computations and data handling [15] and MATLAB (2023b, The Mathworks Inc., Natick, MA) was used for graphical presentations.

## 3 Results

Twelve volunteers were recruited and screened for eligibility. One participant experienced post‐randomization dropout resulting from the inability to obtain intravenous access. Eleven participants completed both scenarios of the trial (Table 1), resulting in 22 individual experimental runs. Participants reached the maximum cooling time in 11 cases and reached the target temperature in the remaining 11 cases.

**FIGURE 1:**
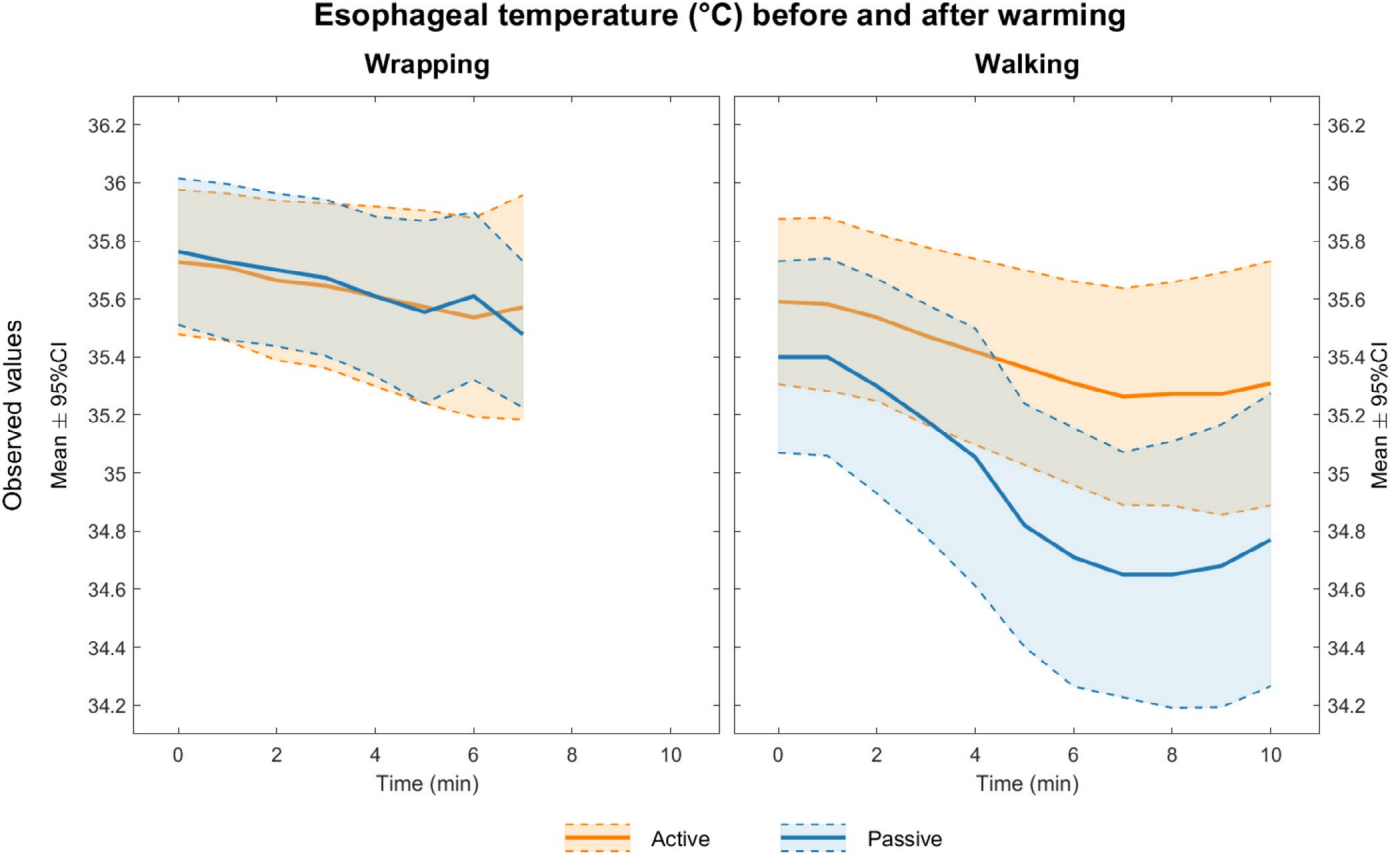
Esophageal temperature before and after warming: this figure shows the mean esophageal temperatures during the wrapping phase (between the cooling and rewarming phases) and the walking phase (after the rewarming phase, as the participants were walking out with minimal support of the tunnel).

The participants experienced a decrease in temperature during the wrapping phase, with a mean esophageal temperature drop of 0.2°C (Figure 1). The participants returned to the pre‐wrapping esophageal temperature after an average of 6 min.

**FIGURE 2:**
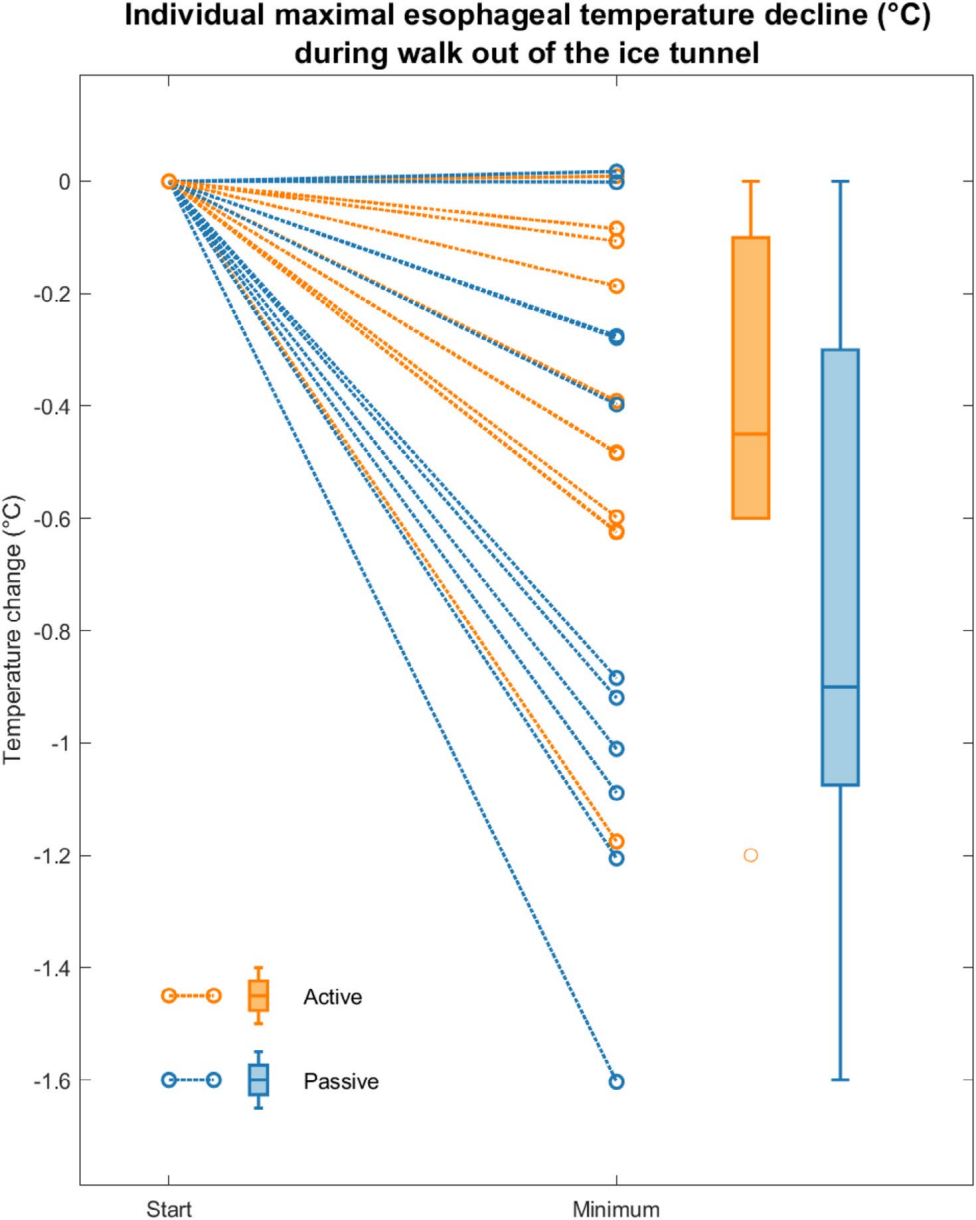
Individual esophageal temperature declines. Individual temperature plots from all participants showing the relative change between the start of the experiment and the lowest recorded esophageal temperature during the minimally supported walk out of the ice tunnel.

Both groups experienced a decrease in esophageal temperature during the 10 min spent walking with minimal support out of the tunnel, with a mean drop of 0.3°C (min 0.0°C, max 1.2°C) in the actively rewarmed group and 0.7°C (min 0.0°C, max 1.6°C) in the passively rewarmed group (Figure 1). The largest individual observed drop of 1.6°C occurred in the passively rewarmed group (Figure 2).

## 4 Discussion

The main finding of this trial was that afterdrop was reduced in the group receiving active external warming. This strengthens the argument that treatment using active external warming may help reduce afterdrop in cases of accidental hypothermia, and that a convective mechanism contributes significantly to afterdrop as suggested by Giesbrecht [16]. A reduction in afterdrop is desirable for several reasons. Decreased afterdrop may reduce the risk of cardiac arrhythmia and cardiac arrest in cold patients. It is also reasonable to believe that a smaller afterdrop results in a quicker return to normothermia, thereby lowering the risk of complications from hypothermia.

Most participants experienced an afterdrop twice during this experiment, both during wrapping and walking out of the ice tunnel. These findings support the circulatory mechanisms of the afterdrop, as both occasions were associated with the physical movement of the participants. The participants had to be moved into the insulating wrapping, increasing peripheral circulation. The movement probably resulted in the return of cold venous blood from the periphery, due to an increased convective heat loss from the core. The esophageal temperature quickly returned to the approximate level it was before the start of the wrapping, when the participant was immobile. At this point, we assume that a substantial temperature gradient between the periphery and core remained in the tissues of the participants. This became apparent at the end of the rewarming phase when the volunteers walked out of the ice tunnel to the recovery tent. The physical effort of walking triggered a more prolonged increase in peripheral circulation than that observed during the wrapping phase, and more cold blood circulated to the periphery before returning to the core. Rescuers should expect that multiple afterdrops may occur when patients are mobilized, especially if patient movement has been limited.

Our findings support the data from a study by Lundgren et al. published in 2009 in which afterdrop size was lower in groups receiving active external warming [17]. This contrasts with the findings of Hurrie et al. in 2020, where no difference in afterdrop was found between groups receiving active or passive warming. Both studies employed a similar pharmacological protocol to inhibit shivering, and both utilized cold water as the cooling medium. However, the physical movement of the participants in these studies appeared to be significantly less than that of the participants in our study when walking to the recovery tent. The faster cooling rates and differences in locomotion may account for the differences in afterdrop characteristics observed in these studies compared to what was found in this experiment.

Although the standardized conditions and use of medications for inhibition of thermoregulation limit the direct clinical impact, the findings of this controlled trial may be transferable to a clinical setting to a certain degree. Shivering thermogenesis is an essential part of passive rewarming, which was not present for the subjects in our control group. Shivering patients may have different heat distribution and consequently different afterdrop characteristics than the non‐shivering healthy participants observed in this study. However, not all patients with hypothermia are capable of shivering. Patients with hypoglycemia, exhaustion, or intoxication may have severely decreased shivering function [18, 19, 20], and the results of this trial may be applicable to patients with these concomitant conditions. Sran et al. studied hypothermic volunteers without inhibition of shivering, finding no significant effect on afterdrop of using active warming devices, compared to shivering alone [21].

There is a risk of inflicting cutaneous burn injuries if too much heat is applied locally, and there are several reported incidents of this [22]. Circulatory changes are not uncommon during the rewarming phase after accidental hypothermia, but the implications for prehospital use of rewarming are unclear [23].

It is possible that the use of meperidine may have influenced the results of this study. Meperidine is an opioid, and will therefore affect vasomotor tone and contribute to vasodilation. As previously discussed, afterdrop is believed to have a significant circulatory component [7]. It is possible that administration of meperidine results in a subsequent increase in cutaneous perfusion, which in turn leads to an increase in the skin surface in contact with the heat source. More contact area may result in higher temperatures in the blood returning from the skin, and consequently a smaller afterdrop.

### 4.1 Heat Debt

External cooling initially increases the heat loss from peripheral tissues. This will increase the temperature gradient between the core and peripheral tissues. Thus, a patient with hypothermia subjected to external cooling will acquire a “heat debt” from core to peripheral tissues, which must be reimbursed during rewarming if external warming methods are used. “Heat debt” is not a new term [24], but it is usually discussed in the context of experimental physiology or human performance in cold environments [25, 26, 27]. To the best of our knowledge, the role of heat debt in the clinical management of patients with accidental hypothermia has not been discussed.

Preventing further heat loss may not be sufficient to correct the peripheral heat deficit; additional heat has to be endogenously produced or externally provided to reinstate a stable heat gradient. The heat gradient between the core and peripheral tissues can be reduced because of the redistribution of heat from the core, with or without supplemental heat provided by external warming. The resulting reduction in the core temperature can be observed as an afterdrop. Active external warming can help rewarm cooled peripheral tissues, thereby reducing the demand for heat from the core and consequently reducing the magnitude of afterdrop. Our results indicates that active warming measures contributed to reducing the peripheral heat debt, resulting in a less severe afterdrop.

## 5 Limitations

The experiment was primarily designed to evaluate the effect of the esophageal temperature rewarming rate, and baseline differences may have limited the findings of this study. For example, recording temperatures more frequently than once every minute could be beneficial. The power calculation was also performed for the original trial.

The short timeframe is a limitation, as data after 10 min could not be compared due to various warming techniques in the recovery tent. Participants were assigned to sleeping bags or saunas based on esophageal temperature, logistics, availability, and personal preferences.

Thermoregulatory suppression increases standardization as it removes the large inter‐individual differences in shivering thresholds and shivering capacity. However, this limits the validity of our findings to non‐shivering patients, as shivering may affect heat gradients and afterdrop. These findings may not be valid for more severe hypothermia, as the degree of hypothermia could potentially influence afterdrop characteristics.

The use of a transnasal probe for temperature measurements carries the risk of misplacement of the probe, as placement of the probe was not verified in all participants.

## 6 Conclusion

This trial demonstrates that additional active external warming contributes to a reduced afterdrop compared with using only passive warming measures, and that victims of hypothermia may experience more than one episode of afterdrop until a stable thermal gradient is re‐established between the core and peripheral tissues. The findings support the use of active external warming in accidental hypothermia.

## Author Contributions

S.M., A.M.H., G.B., Ø.Ø., Ø.W., J.A., G.S., and Ø.T. contributed to the study design. S.M. enrolled participants. S.M., A.M.H., N.B., G.B., Ø.W., J.A., and Ø.T. conducted the experiments and collected data. S.M. processed the data with A.M.H., Ø.W., and J.A. J.A. performed the statistical analyses. S.M. wrote the manuscript, and A.M.H., N.B., G.B., Ø.Ø., Ø.W., J.A., G.S., and Ø.T. critically revised it. Editage language editing service was used to improve the language quality. All authors read and approved the final manuscript. Large language models (LLMs) were not used for any purpose in this article.

## Funding

This work was supported by the Norwegian Air Ambulance Foundation.

## Ethics Statement

The Norwegian Regional Ethics Committee for Medical and Health Research (2024/714469) REK South‐East C and the Data Protection Officer of Haukeland University Hospital approved the original trial, which was registered at ClinicalTrials.gov (NCT 06342726).

## Consent

Written informed consent was obtained from all the participants.

## Conflicts of Interest

The authors declare no conflicts of interest.

## Supporting information


**Data S1:** Supporting Information.

**Data S2:** CONSORT 2025 checklist.

**Data S3:** Figure 1: CONSORT 2025 Flow Diagram.

## Data Availability

The data that support the findings of this study are available on request from the corresponding author. The data are not publicly available due to privacy or ethical restrictions.
