# Supplementary material for: Reduction of Afterdrop by Using Active External Warming During Treatment of Accidental Hypothermia—A Randomized, Crossover Trial
Source: Acta Anaesthesiol Scand. 2025 Dec 1;70(1):e70162. doi: 10.1111/aas.70162 (PMC12668870; doi:10.1111/aas.70162)
Supplement: Supplementary file 1 — Data S1: Supporting Information. [file AAS-70-0-s002.docx]

**Inclusion criteria**

*Age: Over 18 years old*

*General health: Overall healthy*

*BMI: Below* 30 kg/m^2^ but above 18.5 kg/m^2^

*Informed consent: Able to receive, understand and consent to partake in the experiment based on information provided about the experiment*

**Exclusion criteria**

*Allergies: Known allergy to Meperidine or Buspirone*

*Concomitant use of any of the following medications:* MAO-inhibitors, SSRI, Ritonavir, cimetidine, chlorpromazine, phenytoin, erythromycin, itraconazole, anti-platelet agents, Apomorphine.

*Pregnancy*

*Neurologic conditions:* Recent head trauma, high ICP or epilepsy

*General health:* Reduced liver- or kidney function, respiratory failure, cardiac arrhythmias or prolonged QTc, BPH, acute glaucoma, myasthenia gravis or previous cold injury/frost bite.
