# Supplementary material for: Reduction of Afterdrop by Using Active External Warming During Treatment of Accidental Hypothermia—A Randomized, Crossover Trial
Source: Acta Anaesthesiol Scand. 2025 Dec 1;70(1):e70162. doi: 10.1111/aas.70162 (PMC12668870; doi:10.1111/aas.70162)
Supplement: Supplementary file 3 — Data S3: Figure 1: CONSORT 2025 Flow Diagram. [file AAS-70-0-s001.docx]

**Figure 1: CONSORT 2025 Flow Diagram**

Flow diagram of the progress through the phases of a randomised trial of two groups (that is, enrolment, intervention allocation, follow-up, and data analysis)

Analysis

Analysed for primary outcome (n= 11)

Excluded from analysis (give reasons) (n=0 )

Discontinued intervention (give reasons) (n=0 )

Lost to follow-up for primary outcome (give reasons) (n= 0):

Discontinued intervention(give reasons) (n=0 )

Lost to follow-up for primary outcome (give reasons) (n= 0):

Excluded (n=1 )

Not meeting inclusion criteria (n= )

Declined to participate (n= )

Other reasons (n=1 )

Randomised (n= 11)

Allocation

Follow-Up

Allocated to intervention (n=11 )

Received allocated intervention (n= 11)

Did not receive allocated intervention (give reasons) (n= )

Allocated to intervention (n= 11)

Received allocated intervention (n= 11)

Did not receive allocated intervention (give reasons) (n= )

Enrolment

Assessed for eligibility (n= 12)

Analysed for primary outcome (n=11 )

Excluded from analysis (give reasons) (n=0 )
